# The effect of the very low dosage diltiazem on tacrolimus exposure very early after kidney transplantation: a randomized controlled trial

**DOI:** 10.1038/s41598-022-18552-7

**Published:** 2022-08-21

**Authors:** Teerada Susomboon, Yotsaya Kunlamas, Somratai Vadcharavivad, Attapong Vongwiwatana

**Affiliations:** 1 Department of Pharmacy Practice, Faculty of Pharmaceutical Sciences, Chulalongkorn University, Bangkok, 10330 Thailand; 2 Department of Medicine, Faculty of Medicine Siriraj Hospital, Mahidol University, Bangkok, 10700 Thailand

**Keywords:** Translational research, Genetics, Nephrology

## Abstract

The objective of this study was to assess the effect of the very low dosage of diltiazem on tacrolimus exposure during the first week post-kidney transplantation, among cytochrome P450 (CYP) 3A5 expressers who did not receive diltiazem (EXplb), CYP3A5 expressers who received the very low dose diltiazem (EXdtz), CYP3A5 nonexpressers who did not receive diltiazem (NEplb), and CYP3A5 nonexpressers who received the very low dose diltiazem (NEdtz). Forty kidney recipients who receive tacrolimus-based immunosuppressive regimen were randomly assigned, with stratification on the *CYP3A5* genotypes, to receive either diltiazem 30 mg every 12 h or a matched placebo. The observed median dose-adjusted area under the 12-h curve of tacrolimus concentration (AUC/D) at day 7 post-transplantation was lowest in the EXplb group followed by EXdtz, NEplb, and NEdtz at 34.9, 43.6, 49.4, and 71.1 ng*h/mL per mg, respectively. A Kruskal–Wallis test showed a significant difference in the mean ranks of AUC/D among groups. Significant differences between EXplb and NEplb, and between EXplb and NEdtz were demonstrated, whereas no sufficient evidence of significant differences was detected between the other pairs. In conclusion, coadministration of diltiazem 30 mg twice daily may be advantageous for increasing tacrolimus exposure early after kidney transplantation among CYP3A5 expressers.

## Introduction

Tacrolimus, a potent calcineurin inhibitor (CNI), is an immunosuppressive agent widely used for the prevention of acute allograft rejection in kidney transplant recipients (KTRs)^1^. The role of tacrolimus as an essential component of immunosuppressive protocols has been proven^2–4^. Most commonly used in combination with antimetabolite mycophenolate and glucocorticoids, tacrolimus has become the backbone of the primary maintenance immunosuppressive regimen used after kidney transplantation (KT)^5^.

Optimally balanced immunosuppressive therapy is vital for preventing acute rejection, deterioration of kidney graft function, and other unwanted outcomes. Under-immunosuppression increases the risk of cellular and antibody-mediated rejections, and kidney allograft loss, whereas overexposure to immunosuppressant increases the risks of dose-related adverse effects, infections, and other unwanted events^6^. Tacrolimus clinical use is complicated by its narrow therapeutic index, time-varying pharmacokinetics, and wide variation between individuals in systemic drug exposure achieved by a given dose^1,5,7^.

Tacrolimus is a substrate of cytochrome P450 (CYP) 3A microsomal oxidase enzyme system and P-glycoprotein (P-gp). CYP3A5, which is polymorphic expressed, is identified as the principal enzyme responsible for tacrolimus metabolism^8–10^. A single nucleotide polymorphism in the gene encoding for the CYP3A5 enzyme (rs776746), which results in either the absence or a pronounced reduction in CYP3A5 expression, significantly influences tacrolimus exposure^5,8^. To achieve the same target range of tacrolimus blood concentrations, CYP3A5 expressers (CYP3A5*1/*1 and *1/*3) require a higher tacrolimus dosage than nonexpressers (CYP3A5*3/*3)^8,10^.

The risk of immunological complications is generally highest during the early period after KT. Adequate immunosuppression is crucial during this critical period since lower tacrolimus exposure at approximately one week post-KT has been associated with subsequently higher rates of acute rejection^11–14^. Even though therapeutic drug monitoring of tacrolimus is recognized to be an essential component of immunosuppressive therapy in KTRs and routinely implemented in clinical practice for the personalization of tacrolimus dosage to maintain its efficacy and minimize the consequences of overexposure of the drug, therapeutic drug monitoring is of limited value during the early days after KT given that a steady state of tacrolimus pharmacokinetics has not yet been reached. With the same starting tacrolimus dose and therapeutic drug monitoring scheme, the proportion of KTRs achieving tacrolimus concentrations during the first week was significantly lower in CYP3A5 expressers in comparison with nonexpressers and episodes of acute rejection occurred earlier in expressers^15^.

The antihypertensive diltiazem, a non-dihydropyridine calcium-channel blocker (CCB), inhibits activities of CYP3A metabolism and P-gp efflux pump^16,17^. Diltiazem co-administration has been suggested as one of the possible strategies to allow therapeutic tacrolimus levels to be achieved at a lower tacrolimus dosage^18^. Significantly higher dose-adjusted trough concentrations of tacrolimus (C0/D) on day 7 after KT were reported in CYP3A5 expressers who received co-administered diltiazem in a dosage of 30 mg three times daily compared to those who did not receive diltiazem^19^.

Whether or not co-administration of diltiazem in a very low dosage of 30 mg twice daily affects tacrolimus pharmacokinetics during the very early period post-KT amongst CYP3A5 expressers and nonexpressers is questionable. This study was conducted to evaluate the influence of the very low dose diltiazem and *CYP3A5* genetic polymorphisms on tacrolimus exposure in adult Thai KTRs in the first week after transplantation by comparing dose-adjusted area under the 12-h curve of tacrolimus concentration (AUC/D) at day 7 post-KT and the proportions of patients with a markedly subtherapeutic or supratherapeutic tacrolimus trough concentration (C0) at day 3 post-KT, among CYP3A5 expressers who did not receive diltiazem (EXplb), CYP3A5 expressers who received a very low dose diltiazem (EXdtz), CYP3A5 nonexpressers who did not receive diltiazem (NEplb), and CYP3A5 nonexpressers who received a very low dose diltiazem (NEdtz).

## Methods

This prospective, randomized, double-blind, placebo-controlled study was carried out in four parallel groups of KTRs. Eligible KTRs were randomly assigned to receive either oral, immediate-release diltiazem (Ditizem®; Siam Pharmaceutical, Bangkok, Thailand) 30 mg every 12 h or physically identical matched placebo, which was started on the day of KT (day 0), in a 1:1 ratio according to a sequence of computer-generated random schedule in permuted blocks of 4 with stratification on the *CYP3A5* genotypes (CYP3A5 expressers and nonexpressers) by nQuery program version 4.0 (Statistical Solutions Ltd), Cork, Ireland. The randomization schedule was concealed in sequentially numbered, sealed, opaque envelopes, and kept in a locked cabinet in the central pharmacy of Siriraj Hospital. Envelopes were opened sequentially only after enrollment of each participant. The diltiazem and placebo containers, which were issued with a medication number and assigned to consecutive patients in a sequential order, were tamper-proof, equal in weight, and similar in appearance. A pharmacist not involved in the care of the study patients did the randomization, distributed the study agents, and held the trial codes, which were disclosed after the study. Physicians, nurses, other care providers, and the study patients were unaware of the group allocations.

All adult KTRs (> 18 years old) who received their living related KT at Siriraj Hospital, a university hospital in Thailand, between March 2017 and December 2020 and had been prescribed tacrolimus as their primary immunosuppressive regimen post-KT were eligible to participate. Exclusion criteria included multiple organ transplantation, ABO blood group incompatible KT, cirrhosis, aspartate aminotransferase > 100 U/L, alanine aminotransferase > 120 U/L, hepatitis C viral infection, active gastrointestinal disorder, sign of infections, and blood pressure < 110/70 mmHg. Patients who received treatment with anti-thymocyte immunoglobulin, any investigational drugs, or medications (except for methylprednisolone, prednisolone, and omeprazole) that could significantly interfere tacrolimus pharmacokinetics were also excluded.

Demographic and relevant clinical data were obtained from medical records and the electronic laboratory reported program (Éclair program). All transferred data were verified and validated.

### DNA extraction and genotyping

After recruitment, venous blood samples were collected into tubes containing EDTA. Genomic DNA was extracted from the peripheral blood leukocytes using a Puregene Blood Kit (Qiagen®). The concentration of extracted DNA was measured using a Nanodrop2000 spectrophotometer (Thermo Scientific, Waltham, MA) and diluted to 20 ng/μL for genotyping analysis. Genotyping of *CYP3A5* (6986A>G, rs776746, assay ID:C_26201809_30) was assessed using 2 μL of genomic DNA samples (20 ng/μL), TaqMan® Genotyping assay (20×) 1 μL, TaqMan® Universal PCR master mix (2×) 10 μL, distilled water 7 μL (total 20 μL). TaqMan Drug Metabolism Genotyping Assay reactions were run with the following thermal cycling profile: 95 °C for 10 min, followed by 50 cycles at 95 °C for 15 s and 60 °C for 90 s. Cycling and allelic discrimination analysis was performed using the QuantStudioTM 3 Real-Time (Applied Biosystems®).

KTRs who are either heterozygous or homozygous carriers of the CYP3A5*1 allele were classified as CYP3A5 expressers and those who are homozygous carriers of the CYP3A5*3 allele were classified as CYP3A5 nonexpressers.

### Immunosuppressive drug regimen and other medication use

All participants received oral, immediate-release tacrolimus (Prograf®; Astellas, Kerry, Ireland). According to Siriraj Hospital protocol, the initial dose (0.05 mg/kg every 12 h) of tacrolimus was started 2 days before KT (day − 2) and subsequently adjusted after KT based on efficacy and toxicity and to maintain morning trough concentrations of 7 to 10 ng/mL in standard risk patients and 10 to 12 ng/mL in high-risk patients during the first week after transplantation. Participants also received either mycophenolate mofetil (Cellcept®; F Hoffmann-La Roche Ltd, Basel, Switzerland or Immucept®; Intas Pharmaceuticals Ltd., Gujarat, India) or mycophenolate sodium (Myfortic®; Novartis Pharma Stein, Switzerland for Novartis AG, Basel, Switzerland) at the dosage equivalent to 2 g/day of mycophenolate mofetil. A tapered corticosteroid regimen was administered consisting of an intravenous bolus of 500 mg of methylprednisolone (Solu-medrol®; Novartis Pharma Stein, Switzerland for Novartis AG, Basel, Switzerland) on day 0, 250 mg on day 1, 125 mg on day 2 followed by 40 mg/day of prednisolone on day 4 after KT. The subsequent prednisolone doses were gradually tapered thereafter. Induction therapy with basiliximab (Simulect®; Novartis Pharma AG, Basel, Switzerland) was allowed.

Either 30 mg diltiazem or matched placebo capsule was given every 12 h at the same time as tacrolimus dose. All patients received a fixed dose of omeprazole 40 mg/day. Other antihypertensive drugs, except for those significantly affected with tacrolimus pharmacokinetics, were allowed at the discretion of physician according to standard of care.

### Tacrolimus concentration analysis

On day 7 post-KT, after the tacrolimus dosage was stable for at least 48 h, venous blood samples (5 mL) were collected at pre-dose and 1, 2, 4, 6, 8 and 12 h after the morning dose of tacrolimus. Samples were collected in EDTA tubes and stored at 4 degrees Celsius until analysis. Whole blood tacrolimus concentrations were determined within the same day of blood collection by using a chemiluminescent microparticle immunoassay method with ARCHITECT system® i2000 (Abbott, Abbott Diagnostic, USA). The method was linear in the concentration range up to 30 ng/mL, the limits of quantification were 0.8 ng/mL, and the coefficient of variation of within run precision and between run precision were less than 10%. Mean recovery was 100 ± 10% according to the manufacturer’s information^20^.

### Outcome measures

The primary outcome measure was AUC/D at day 7 post-KT. Secondary outcomes were the proportions of patients with a markedly subtherapeutic (< 5 ng/mL) or supratherapeutic (> 15 ng/mL) tacrolimus C0 and measured systolic and diastolic blood pressures on days 3 and 7 post-KT.

The area under the 12-h curve of tacrolimus concentration (AUC) was calculated by linear logarithmic trapezoidal method. For calculation of AUC/D and C0/D, values of AUC and C0 were dose-adjusted by dividing the observed values by the dose recorded on the day of blood collection. Early-morning blood pressures were measured on the same day of blood collection for pharmacokinetic analysis by a ward nurse as part of a routine examination, during rest in a seated position with a calibrated and well-maintained automatic sphygmomanometer. Delayed graft function (DGF) was defined as failure of the kidney graft to function immediately, with the need for dialysis in the first 7 days post-KT. Episode of biopsy-proven acute rejection (BPAR) during 3 months post-KT was diagnosed based on histologic findings in indication kidney graft biopsy according to Banff 2015 criteria.

### Statistical analysis

The distribution of continuous data was evaluated by Shapiro–Wilk test, and subsequently, parametric tests or nonparametric tests were applied as appropriate. Normally distributed continuous data are presented as mean ± standard deviation (SD) and non-normally distributed continuous data as medians with interquartile ranges (IQR), unless stated otherwise. Counts and percentages are expressed for categorical data.

Genotype frequencies of the polymorphisms were tested for the deviations from Hardy–Weinberg equilibrium using appropriate chi-square testing. The analysis of variance (ANOVA) or Kruskal–Wallis test was used to compare continuous data. For the comparison of AUC/D among the four study groups, eta squared, a measure of effect size for ANOVA was calculated if appropriate; otherwise, epsilon squared, a measure of effect size for Kruskal–Wallis, was computed based on the H statistic, which was adjusted for ties. For ANOVA, the Bonferroni correction was carried out for multiple pairwise testing when variances are homogenous and the Dunnett’s T3 test was applied when variances are not homogenous. For Kruskal–Wallis test, the Bonferroni procedure was performed for multiple pairwise comparison. Fisher’s exact test was used to compare proportions among groups. Bivariate correlations between continuous variables were assessed by either Pearson’s correlation test or Spearman’s rank correlation test.

Power calculation was performed. A required total sample size of at least 40 was estimated by G*Power 3.1.9.2 software, Heinrich-Heine-Universität, Düsseldorf, Germany (with an effect size of 0.50, a power of 0.9, an estimated dropout rate of 50%, and a significant level of 0.05).

All statistical tests were conducted against a two-sided alternative hypothesis employing a significant level of 0.05. All analyses were performed using IBM SPSS statistics 28 (IBM, Bangkok, Thailand).

### Ethics

This study was conducted in compliance with the provisions of the Declaration of Helsinki, Declaration of Istanbul, and Good Clinical Practice Guidelines, approved by the Siriraj Institutional Review Board, Faculty of Medicine, Siriraj Hospital, Mahidol University (approval number Si 721/2016), and registered in the Thai Clinical Trials Registry (TCTR20170206005) with the full date of first registration on 06/02/2017. All the patients provided written informed consent before enrollment.

## Results

**Figure 1:**
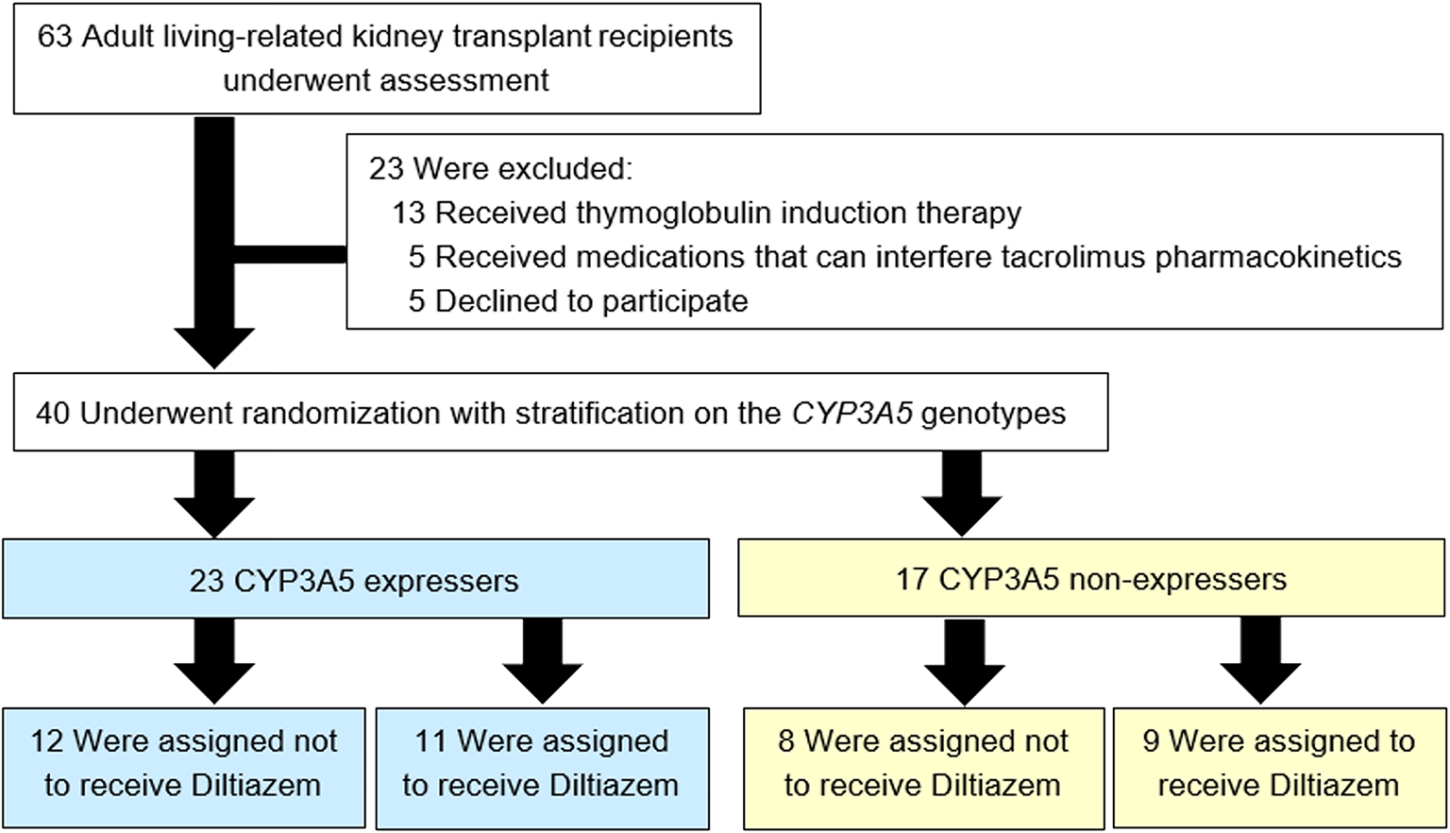
Flow diagram of patient enrollment.

In this study, 63 living related KTRs were assessed for eligibility. Thirteen received anti-thymocyte globulin for induction therapy, five received any medication that could significantly interfere tacrolimus pharmacokinetics, and five declined to participate. The study population thus comprised a total of 40 KTRs (Fig. 1).

The observed allele frequencies of CYP3A5*1 and CYP3A5*3 were 36.2% and 63.8%, respectively. The genotype frequencies were in accordance with the Hardy–Weinberg equilibrium (chi-square = 0.259, p = 0.611). The CYP3A5*1/*1, *1/*3, or *3/*3 genotypes were detected in 6 (15.0%), 17 (42.5%), and 17 (42.5%) patients, respectively. Twenty-three (57.5%) were identified as CYP3A5 expressers, and 17 (42.5%) were nonexpressers.

Demographic and clinical characteristics on day 7 post-KT were well balanced among four randomly assigned groups as summarized in Table 1. Of 40 KTRs, the overall mean ± SD of tacrolimus dosage on day 7 post-KT was 6.7 ± 2.8 mg/day. On day 7, the mean ± SD of tacrolimus AUC was 153.6 ± 33.2 ng*h/mL (range 96.9–232.8 ng*h/mL), with the corresponding C0 of 8.4 ± 2.1 ng/mL (range 5.0–13.2 ng/mL). The median (IQR) of AUC/D and C0/D were 44.9 (37.1, 65.3) ng*h/mL per mg and 2.5 (1.8, 3.7) ng/mL per mg, respectively. A moderate positive correlation between AUC and C0 was observed (Pearson correlation coefficient, *r* = 0.617; p < 0.001).

**Table 1:** Patient characteristics on day 7 after kidney transplantation.

Characteristics	CYP3A5 expressers		CYP3A5 non-expressers	
	Placebo (n = 12)	Diltiazem (n = 11)	Placebo (n = 8)	Diltiazem (n = 9)
**Female, n (%)**	3 (25.0)	6 (54.5)	4 (50.0)	2 (22.2)
**Age, years**	48 (44, 56)	34 (30, 58)	42 (35, 58)	38 (33, 57)
**Body weight, kg**	60.8 ± 13.8	51.7 ± 10.4	59.3 ± 13.4	55.7 ± 11.0
**Height, m**	1.64 (1.60, 1.70)	1.60 (1.55, 1.70)	1.58 (1.55, 1.70)	1.64 (1.56, 1.67)
**Cause of ESRD, n (%)**				
Diabetes Mellitus	3 (25.0)	0 (0)	2 (25.0)	2 (22.2)
Hypertension	1 (8.3)	1 (9.1)	0 (0)	0 (0)
Chronic glomerulonephritis	0 (0)	3 (27.3)	0 (0)	1 (11.1)
IgA nephropathy	2 (16.7)	1 (9.1)	1 (12.5)	0 (0)
Other	1 (8.3)	1 (9.1)	3 (37.5)	1 (11.1)
Unknown	5 (41.7)	5 (45.5)	2 (25.0)	5 (55.6)
**Donor age, years**	35 (24, 44)	33 (30, 48)	38 (23, 41)	38 (32, 41)
**Female donor, n (%)**	8 (66.7)	7 (63.6)	3 (37.5)	5 (55.6)
**RRT before KT, n (%)**				
Preemptive	5 (41.7)	2 (18.2)	0 (0)	2 (22.2)
Hemodialysis	5 (41.7)	8 (72.7)	6 (75.0)	7 (77.8)
Peritoneal dialysis	2 (16.7)	1 (9.1)	2 (25.0)	0 (0)
**Dialysis vintage, months**	3 (0, 17)	15 (1, 22)	14 (10, 32)	15 (1, 46)
**Number of HLA mismatch**	3.0 (0.5, 4.5)	3.0 (2.0, 5.0)	2.5 (2.0, 3.0)	3.0 (2.5, 4.5)
**Presence of pre-transplant HLA antibodies**^**a**^**, n (%)**				
DSA	0 (0)	0 (0)	0 (0)	0 (0)
Non-DSA	5 (41.7)	2 (18.2)	3 (37.5)	2 (22.2)
**Serum creatinine, mg/dL**	1.19 (1.04, 1.67)	1.10 (0.97, 1.53)	1.02 (0.78, 1.30)	1.13 (0.96, 1.60)
**Hemoglobin, g/dL**	11.0 ± 2.0	11.3 ± 2.2	10.5 ± 1.7	10.8 ± 1.4
**Albumin, g/dL**	3.6 (2.9, 3.8)	3.7 (3.4, 3.9)	3.3 (3.2, 3.4)	3.7 (3.5, 3.9)
**Aspartate transaminase**^**b**^**, U/L**	17 (12, 33)	15 (10, 22)	17 (14, 21)	17 (10, 23)
**Alanine transaminase**^**b**^**, U/L**	17 ± 8	15 ± 4	13 ± 7	19 ± 8
**Total bilirubin**^**b**^**, mg/dL**	0.43 ± 0.12	0.39 ± 0.16	0.43 ± 0.13	0.48 ± 0.20
**Direct bilirubin**^**b**^**, mg/dL**	0.15 (0.13, 0.19)	0.13 (0.11, 0.20)	0.15 (0.13, 0.17)	0.19 (0.10, 0.30)

Values are expressed as count (%), mean ± SD, or median (interquartile range) for comparison reasons.

*ESRD* end stage renal disease, *DSA* donor-specific antibody, *HLA* human leukocyte antigen, *KT* kidney transplantation, *RRT* renal replacement therapy.

^a^Detection by single-antigen bead analysis, with mean fluorescence intensity of greater than or equal to 1500 (LABScreen Luminex kits, One Lambda, Canoga Park, CA, USA).

^b^Data on day − 2 before kidney transplantation.

**Figure 2:**
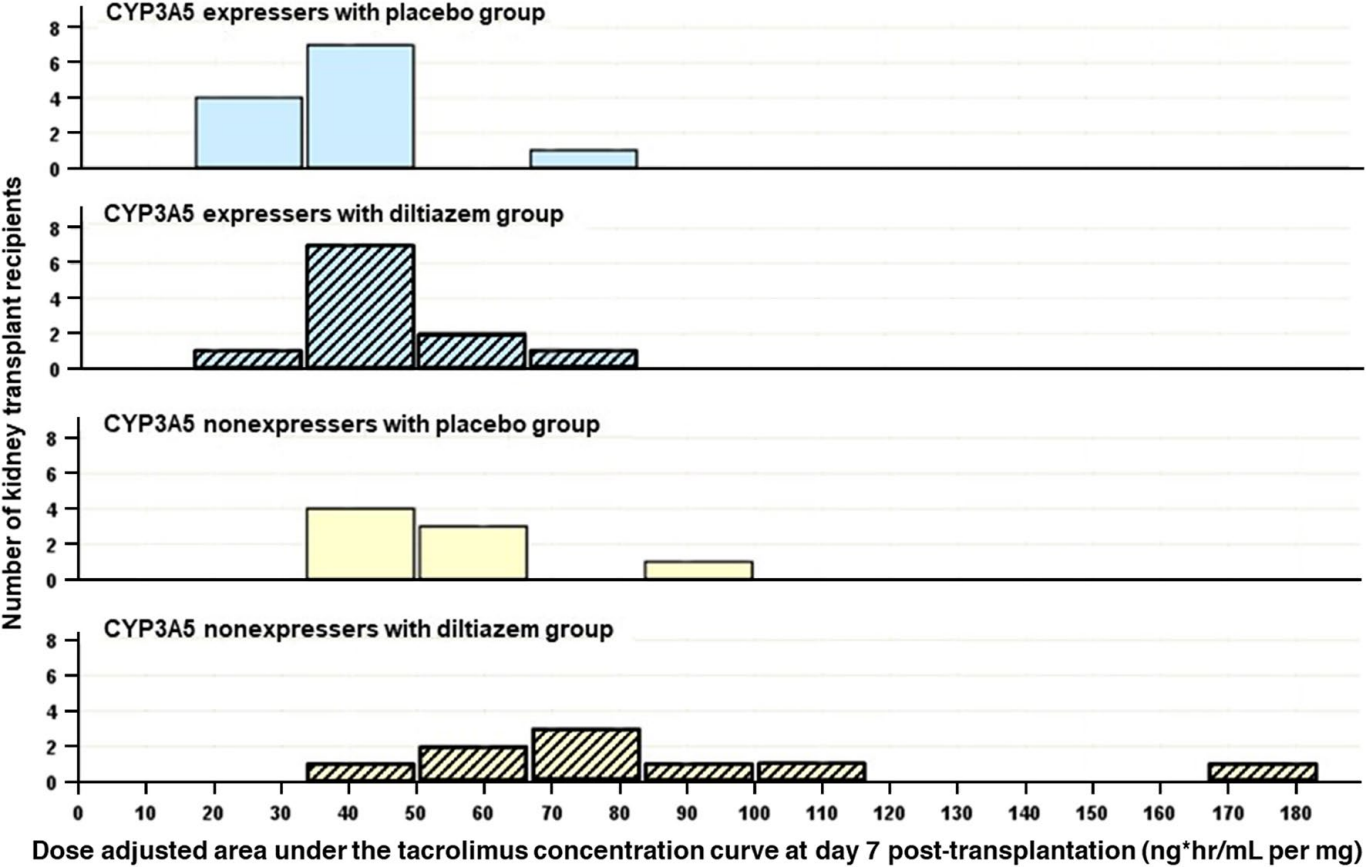
Dose-adjusted area under the 12-h curve of tacrolimus concentration at day 7 post-kidney transplantation.

The observed median AUC/D at day 7 was lowest in the EXplb group followed by EXdtz, NEplb, and NEdtz at 34.9, 43.6, 49.4, and 71.1 ng*h/mL per mg, respectively as presented in Table 2. The frequency distributions of AUC/D in each study group were displayed in Fig. 2. A Kruskal–Wallis H test showed a significant difference in the mean ranks of AUC/D among the four groups (H = 19.704, df = 3, p < 0.001), with a large effect size (epsilon squared = 0.505). To identify significant differences between specific groups, pairwise comparisons with the Bonferroni correction for multiple testing were carried out. The mean rank for EXplb is significantly lower than both the mean rank for NEplb (adjusted p = 0.026) and significantly lower than the mean rank for NEdtz (adjusted p < 0.001), whereas the differences between the other pairs were not statistically significant as shown in Table 3.

**Table 2:** Tacrolimus dose, concentration, and area under the 12-h concentration curve.

	CYP3A5 expressers		CYP3A5 non-expressers		P value
	Placebo (n = 12)	Diltiazem (n = 11)	Placebo (n = 8)	Diltiazem (n = 9)	
**2 days before transplantation**					
Daily dose, mg/day	6.0 (6.0, 6.8)	5.0 (5.0, 6.0)	5.5 (5.0, 8.0)	6.0 (5.0, 6.5)	0.414^a^
Daily dose, mg/kg/day	0.10 (0.08, 0.10)	0.10 (0.09, 0.11)	0.10 (0.09, 0.11)	0.10 (0.09, 0.11)	0.820^a^
**Day 1 post-transplantation**					
Daily dose, mg/day	6.0 (6.0, 7.0)	5.0 (5.0, 7.0)	6.0 (5.0, 7.8)	6.0 (4.5, 6.0)	0.266^a^
Daily dose, mg/kg/day	0.10 ± 0.02	0.11 ± 0.02	0.10 ± 0.03	0.09 ± 0.02	0.396^b^
Dose, mg/kg	0.05 ± 0.01	0.06 ± 0.01	0.05 ± 0.02	0.05 ± 0.01	0.396^b^
C0, ng/mL	7.0 ± 3.2	10.9 ± 3.7	12.6 ± 7.2	13.1 ± 7.4	0.055^b^
C0/D, ng/mL per mg	2.2 (1.3, 2.8)	3.3 (2.6, 4.5)	3.8 (2.0, 5.0)	3.8 (2.7, 7.2)^c^	0.026^a^
C0/Daily dose, ng/mL per mg/day	1.1 (0.6, 1.4)	1.6 (1.3, 2.3)	1.9 (1.0, 2.5)	1.9 (1.3, 3.6)^c^	0.026^a^
**Day 3 post-transplantation**					
Daily dose, mg/day	7.5 ± 1.6	5.4 ± 1.7	5.1 ± 2.1	4.4 ± 2.4^c^	0.005^b^
Daily dose, mg/kg/day	0.12 ± 0.04	0.10 ± 0.04	0.09 ± 0.04	0.08 ± 0.05	0.092^b^
Dose, mg/kg	0.06 ± 0.02	0.05 ± 0.02	0.04 ± 0.02	0.04 ± 0.03	0.092^b^
C0, ng/mL	6.2 (4.4, 8.0)	9.3 (7.0, 10.4)	9.4 (8.8, 12.4)^c^	11.9 (8.8, 13.4)^c^	< 0.001^a^
C0/D, ng/mL per mg	1.8 (1.1, 2.0)	3.7 (2.4, 4.5)^c^	3.4 (2.7, 5.8)^c^	6.4 (4.1, 8.8)^c^	< 0.001^a^
C0/Daily dose, ng/mL per mg/day	0.9 (0.6, 1.0)	1.8 (1.2, 2.2)^c^	1.7 (1.4, 2.9)^c^	3.2 (2.0, 4.4)^c^	< 0.001^a^
**Day 7 post-transplantation**					
Daily dose, mg/day	8.8 (8.0, 11.8)	6.0 (5.5, 8.0)^c^	6.2 (5.0, 6.9)^c^	3.5 (2.8, 6.2)^c^	< 0.001^a^
Daily dose, mg/kg/day	0.17 ± 0.08	0.12 ± 0.04	0.10 ± 0.03	0.08 ± 0.04^d^	0.004^b^
Dose, mg/kg	0.08 ± 0.04	0.06 ± 0.02	0.05 ± 0.01	0.04 ± 0.02^d^	0.004^b^
C0, ng/mL	9.2 ± 1.6	7.4 ± 2.1	8.6 ± 2.5	8.4 ± 1.8	0.188^b^
C0/D, ng/mL per mg	2.0 (1.4, 2.5)	1.9 (1.8, 3.2)	2.8 (2.5, 3.4)	4.2 (2.8, 6.0)^c^	0.009^a^
C0/Daily dose, ng/mL per mg/day	1.0 (0.7, 1.2)	1.0 (0.9, 1.6)	1.4 (1.2, 1.7)	2.1 (1.4, 3.0)^c^	0.009^a^
AUC, ng*h/mL	168.8 ± 32.2	138.1 ± 29.6	158.8 ± 40.7	147.8 ± 25.5	0.144^b^
AUC/D, ng*h/mL per mg	34.9 (31.6, 38.5)	43.6 (37.0, 50.5)	49.4 (43.0, 62.9)^c^	71.1 (54.9, 100.7)^c^	< 0.001^a^

Values are expressed as mean ± SD or median (interquartile range) for comparison reasons.

*AUC* area under the concentration over 12-h curve, *AUC/D* dose-adjusted area under the concentration over 12-h curve, *C0* trough concentration, *C0/D* dose-adjusted trough concentration.

^a^Kruskal–Wallis test.

^b^Analysis of variance.

^c^P < 0.05 versus CYP3A5 expressers with placebo group (Bonferroni correction for multiple comparisons).

^d^P < 0.05 versus CYP3A5 expressers with placebo group (Dunnett’s T3 correction for multiple comparisons).

**Table 3:** Comparison of tacrolimus dose-adjusted area under the 12-h curve at day 7 post-kidney transplantation among groups.

Group	Mean rank	Kruskal–Wallis H	P value
EXplb (n = 12)	10.2	19.704	< 0.001
EXdtz (n = 11)	18.8		
NEplb (n = 8)	25.4		
NEdtz (n = 9)	32.0		

Pairwise comparison	Mean rank difference	Standard error	P value^a^
EXplb–NEdtz	− 21.83	5.16	< 0.001
EXplb–NEplb	− 15.21	5.34	0.026
EXplb–EXdtz	− 8.65	4.88	0.457
EXdtz–NEdtz	− 13.18	5.25	0.073
EXdtz–NEplb	− 6.56	5.43	1.000
NEdtz–NEplb	− 6.62	5.68	1.000

*EXplb* CYP3A5 expressers with placebo group, *EXdtz* CYP3A5 expressers with diltiazem group, *NEplb* CYP3A5 nonexpressers with placebo group, *NEdtz* CYP3A5 nonexpressers with diltiazem group.

^a^Bonferroni correction for multiple comparisons.

On day 3 post-KT, four KTRs in the EXplb group and one in the EXdtz group had tacrolimus C0 < 5 ng/mL, while none of the NEplb and NEdtz groups had tacrolimus C0 < 5 ng/mL. However, no statistically significant differences of the proportions of KTRs with tacrolimus C0 of < 5 ng/mL or > 15 ng/mL on days 3 and 7 post-KT were found among the four groups as presented in Table 4. Comparable systolic and diastolic blood pressures were observed among the study groups on days 1, 3, and 7 as reported in Table 5. None of the KTRs had hypotensive episode, DGF, suspected rejection, or BPAR during the first week post-KT.

**Table 4:** Proportions of kidney transplant recipients with a markedly subtherapeutic or supratherapeutic tacrolimus trough concentration during the first week after kidney transplantation.

Tacrolimus trough concentration	Total (n = 40)	CYP3A5 expressers		CYP3A5 non-expressers		P value^a^
		Placebo (n = 12)	Diltiazem (n = 11)	Placebo (n = 8)	Diltiazem (n = 9)	
**Day 1 post-transplantation**						
Lower than 5 ng/mL, n (%)	6 (15.0)	4 (33.3)	0 (0)	1 (12.5)	1 (11.1)	0.152
Lower than the target range, n (%)	12 (30.0)	7 (58.3)	1 (9.1)	2 (25.0)	2 (22.2)	0.078
Higher than 15 ng/mL, n (%)	7 (17.5)	0 (0)	1 (9.1)	3 (37.5)	3 (33.3)	0.053
**Day 3 post-transplantation**						
Lower than 5 ng/mL, n (%)	5 (12.5)	4 (33.3)	1 (9.1)	0 (0)	0 (0)	0.078
Lower than the target range, n (%)	10 (25.0)	8 (66.7)	2 (18.2)^b^	0 (0)^b^	0 (0)^b^	< 0.001
Higher than 15 ng/mL, n (%)	1 (2.5)	0 (0)	0 (0)	1 (12.5)	0 (0)	0.200
**Day 5 post-transplantation**						
Lower than 5 ng/mL, n (%)	4 (10.0)	1 (8.3)	1 (9.1)	2 (25.0)	0 (0)	0.491
Lower than the target range, n (%)	18 (45.0)	7 (58.3)	7 (63.6)	2 (25.0)	2 (22.2)	0.158
Higher than 15 ng/mL, n (%)	0 (0)	0 (0)	0 (0)	0 (0)	0 (0)	–
**Day 7 post-transplantation**						
Lower than 5 ng/mL, n (%)	0 (0)	0 (0)	0 (0)	0 (0)	0 (0)	–
Lower than the target range, n (%)	12 (30.0)	1 (8.3)	6 (54.5)	2 (25.0)	3 (33.3)	0.115
Higher than 15 ng/mL, n (%)	0 (0)	0 (0)	0 (0)	0 (0)	0 (0)	–

^a^Fisher’s exact test.

^b^P < 0.05 versus CYP3A5 expressers with placebo group (Bonferroni correction for multiple comparisons).

**Table 5:** Blood pressures, pulse rate, and number of antihypertensive medication use.

	CYP3A5 expressers		CYP3A5 non-expressers		P value
	Placebo (n = 12)	Diltiazem (n = 11)	Placebo (n = 8)	Diltiazem (n = 9)	
**2 days before transplantation**					
Systolic blood pressure (mmHg)	154.5 ± 36.1	149.3 ± 29.8	144.9 ± 16.7	144.0 ± 14.6	0.808^a^
Diastolic blood pressure (mmHg)	84.7 ± 18.5	90.6 ± 17.2	84.8 ± 10.1	80.4 ± 12.8	0.550^a^
Pulse rate (bpm)	77.0 (68.0, 89.0)	79.0 (68.0, 82.0)	85.0 (72.0, 99.0)	70.0 (65.0, 87.0)	0.363^b^
Number of antihypertensive drugs^c^	2.5 ± 1.4	2.4 ± 1.6	2.5 ± 2.3	3.1 ± 1.5	0.813^a^
**Day 1 post-transplantation**					
Systolic blood pressure (mmHg)	150.9 ± 23.1	152.7 ± 15.9	155.6 ± 11.3	151.2 ± 10.3	0.932^a^
Diastolic blood pressure (mmHg)	85.2 ± 18.5	89.2 ± 13.3	90.8 ± 8.4	87.4 ± 14.8	0.853^a^
Pulse rate (bpm)	83.0 (74.5, 99.5)	82.0 (74.0, 86.0)	98.0 (70.5, 103.5)	82.0 (73.0, 86.0)	0.716^b^
Number of antihypertensive drugs^c^	0 (0, 0.8)	0 (0, 1.0)	0 (0, 0)	0 (0, 1.0)	0.646^b^
**Day 3 post-transplantation**					
Systolic blood pressure (mmHg)	148.5 ± 23.3	144.2 ± 14.7	156.2 ± 14.2	146.7 ± 17.1	0.546^a^
Diastolic blood pressure (mmHg)	89.6 ± 18.2	86.8 ± 13.5	94.8 ± 8.1	84.3 ± 14.0	0.491^a^
Pulse rate (bpm)	81.0 ± 17.8	86.6 ± 15.4	88.2 ± 13.8	82.4 ± 7.2	0.657^a^
Number of antihypertensive drugs^c^	0 (0, 2.0)	1.0 (0, 1.0)	1.0 (0, 1.8)	1.0 (0, 1.5)	0.936^b^
**Day 7 post-transplantation**					
Systolic blood pressure (mmHg)	133.4 ± 21.6	131.4 ± 23.2	127.6 ± 14.2	138.2 ± 10.8	0.704^a^
Diastolic blood pressure (mmHg)	78.4 ± 9.5	77.8 ± 13.3	75.0 ± 11.7	81.0 ± 7.3	0.721^a^
Pulse rate (bpm)	82.3 ± 18.4	80.2 ± 11.2	81.0 ± 19.5	81.8 ± 8.6	0.988^a^
Number of antihypertensive drugs^c^	1.0 (0, 2.0)	1.0 (0, 2.0)	0.5 (0, 1.0)	1.0 (0, 2.0)	0.728^b^

Values are expressed as mean ± SD or median (interquartile range) for comparison reasons.

^a^Analysis of variance.

^b^Kruskal–Wallis test.

^c^Not include diltiazem.

During 3 months after transplantation, two (5%) patients had BPAR. One patient in EXdtz group had an antibody-mediated rejection and one in NEplb had an acute cellular rejection. No borderline changes were detected. Two (5%) patients had post-transplant diabetes mellitus. Of these patients, one was in EXplb and the other was in EXdtz. No study patients reported tremor.

## Discussion

This randomized controlled trial was conducted to determine whether or not there is an effect of the very low dose diltiazem on AUC/D of tacrolimus on day 7 post-KT among 40 living related KTRs, of which approximately 58% were CYP3A5 expressers and 42% were CYP3A5 nonexpressers. All participants received tacrolimus in combination with mycophenolate and corticosteroids. Amongst the four randomly assigned groups, a significant effect of the very low dose diltiazem on AUC/D was demonstrated, with a large effect size. When compared among groups with the same genotype, no significant differences between those who received and did not receive the very low dose diltiazem were found both when compared among CYP3A5 expressers groups and among nonexpressers groups. However, it appears that CYP3A5 expressers that did not receive diltiazem coadministration had a significantly lower mean rank of AUC/D than those in both groups of CYP3A5 nonexpressers, whereas the differences between CYP3A5 expressers who received diltiazem and neither of the groups of CYP3A5 nonexpressers were observed, indicating that the very low dose diltiazem affects tacrolimus exposure in CYP3A5 expressers and may reduce tacrolimus dosage requirement in CYP3A5 expressers to achieve the same exposure of the drug in nonexpressers during the first week after kidney transplantation.

In addition, although no statistically significant differences in proportions of KTRs who had markedly subtherapeutic tacrolimus C0 of < 5 ng/mL on day 3 post-KT were found, one-third of CYP3A5 expressers who did not receive diltiazem coadministration had day 3 C0 of < 5 ng/mL compared to approximately one-eleventh of CYP3A5 expressers who received diltiazem and none of nonexpressers had day 3 C0 of < 5 ng/mL. These findings support that coadministration of the very low dose diltiazem of 30 mg twice daily influences tacrolimus exposure during the first week post-KT and may be considered as a strategy for increasing the achievement of therapeutic tacrolimus exposure early after KT among CYP3A5 expressers when the same starting dose as for nonexpressers is given.

A different expression of CYP3A5, the major enzyme responsible for tacrolimus metabolism, is known to cause inter-patient variability of tacrolimus exposure in KTRs. The gene encoding CYP3A5 is polymorphically expressed. A single nucleotide polymorphism involves an A to G transition at position 6986 within intron 3 of the gene has a well-established influence on tacrolimus pharmacokinetics^8^. Heterozygous or homozygous carriers of the CYP3A5*1 produce high levels of full-length *CYP3A5* mRNA and express high levels of functional CYP3A5 protein, while homozygous carriers of the CYP3A5*3 produce very low or undetectable levels of functional CYP3A5 protein^21^. CYP3A5 expressers require a significant higher tacrolimus dosage, approximately 1.5 to twofold, to reach the same target of the drug exposure^15,22–25^.

The frequency of the CYP3A5*1 allele is highly dependent on ethnicity. Compared to Caucasians, the CYP3A5 expresser is more frequently present in Asians and African Americans^22–28^. In this current study, the CYP3A5*1 allele was identified in approximately 55% of participants. The observed CYP3A5*1 and CYP3A5*3 allele frequencies were in Hardy–Weinberg equilibrium and are similar to those reported in Asian populations, but differ from those seen in Caucasians and African Americans.

The different expression of CYP3A5 not only differs tacrolimus concentrations in systemic circulation, but also may influence a patient’s susceptibility to tacrolimus drug interactions. In the present study, the influence of very low dosage diltiazem of 60 mg/day on tacrolimus AUC/D on day 7 post-KT in CYP3A5 expressers not in CYP3A5 nonexpressers was demonstrated. In a similar way, the tacrolimus-sparing effect of a higher dosage of 90 mg/day diltiazem coadministration was observed in CYP3A5 expressers (p = 0.001), but not remarkable in CYP3A5 nonexpressers (p = 0.201) on day 7 post-KT in a previous study by Li et al.^19^.

CCB is generally considered a preferred antihypertensive agent for KTRs^18^. Diltiazem is a non-dihydropyridine CCB which is well-known to be an inhibitor of CYP3A enzyme activity^29,30^. The use of a non-dihydropyridine CCB to minimize CNI dose has been suggested as one of the strategies that may reduce drug costs given that the concomitant use of a CYP inhibitor increases blood levels of tacrolimus, thereby allowing therapeutic blood levels of tacrolimus to be achieved at a lower dose of tacrolimus^18^. Notably, the tacrolimus dose reduction achieved with diltiazem is modest compared to ketoconazole, a commonly used antifungal agent which is known to be a potent CYP3A enzyme inhibitor and has also been suggested as another strategy that may reduce drug costs in KTRs. However, it was mentioned that if ketoconazole coadministration was discontinued abruptly, tacrolimus blood levels may decrease precipitously, which may increase the risk of acute graft rejection. A sudden drop of tacrolimus levels is less likely with non-dihydropyridine CCB usage^18^.

The inhibitory effect of diltiazem on CYP3A and P-gp activities is a plausible explanation for pharmacokinetic interaction between diltiazem and tacrolimus. The metabolism of diltiazem involves N-demethylation, which is catalyzed extensively by CYP3A. Diltiazem N-demethylated metabolites are competitive inhibitors of CYP3A^31,32^. The findings from experimental studies and in vivo in healthy volunteers suggest that the inhibitory effect of diltiazem on CYP3A activity, in both human liver and intestine, is irreversible and the mechanism of CYP3A inhibition caused by diltiazem is primarily by the formation of P-450-iron (II) metabolite complex, which results in a catalytically inactive CYP3A enzyme^16,33,34^. Tacrolimus is extensively metabolized by CYP3A enzymes^8^. The inhibition of CYP3A by diltiazem, therefore, may enhance intestinal absorption and decrease metabolism of tacrolimus with a consequent increase in drug exposure. Moreover, tacrolimus is a substrate of P-gp, an ATP-dependent efflux drug transporter, which is constitutively expressed in normal tissues including the gastrointestinal epithelium, the canalicular membrane of the liver, and the proximal and distal tubule of the kidney^35^. P-gp transports a variety of substrates across cellular membranes and functionally interact with CYP3A in intracellular drug metabolism. In the small intestine, drugs that are CYP3A and P-gp substrates are repeatedly absorbed into enterocytes and pumped out of cells by P-gp, which keeps intracellular drug concentrations within the linear range of the metabolizing capacity of the CYP3A enzymes^36^. Repeated presentation of the substrates to CYP3A enzymes within the intestinal cell decreases the rate of absorption and increases the opportunity of CYP3A-dependent metabolism^36^. Besides its effects as an inhibitor of CYP3A, diltiazem is also a known P-gp inhibitor. The inhibitory effect of diltiazem on intestinal P-gp activity may enhance tacrolimus absorption by blocking efflux transport, decrease metabolism of the drug, thereby, further increasing tacrolimus exposure^8^.

This is the first prospective randomized clinical study that demonstrates the impact of very low dosage of 60 mg/day diltiazem reducing the impact of the *CYP3A5* genetic polymorphisms on tacrolimus exposure during the first week post-KT. Notably, the dose–response relationship of the pharmacokinetic interaction between tacrolimus and diltiazem has been reported in two stable KTRs in a previous study in which the tacrolimus-sparing effect of diltiazem has been observed to be dose-dependent over a diltiazem dosage range of 20–180 mg per day^37^.

In the present study, all KTRs received a bodyweight-based starting dose of tacrolimus. Although CYP3A5 expressers tended to have a markedly subtherapeutic tacrolimus trough concentration more often during the first 3 days post-KT, nonexpressers tended to have a markedly supratherapeutic trough concentration more often on day 1 post-KT, extensive therapeutic drug monitoring is performed and rapidly corrected any extreme tacrolimus concentrations and none of the study patients had their tacrolimus C0 outside the range of 5–15 ng/mL on day 7 post-KT.

A more appropriate tacrolimus starting dose would limit the time a patient is outside the target levels in the critical early days after transplantation. Among several approaches to reduce the risk of tacrolimus underexposure early after transplantation, the Clinical Pharmacogenetics Implementation Consortium recommends to individualize initial tacrolimus dosing guided in the *CYP3A5* genotyped patients^38^. However, this strategy has not yet been widely implemented in clinical practice. To reduce the risk of underexposure with tacrolimus during the very early period after KT, the initiation of tacrolimus therapy 2 days prior to transplantation has been used in the present study. Of note, more than half of KTRs in EXplb group had their tacrolimus C0 lower than the therapeutic target level on days 1 and 3 post-KT, although the statistically significant difference of the proportions of EXplb in whom tacrolimus C0 was lower than the target level from the other three groups was found only on day 3. This finding re-emphasizes the need for close therapeutic drug monitoring during the high-risk early period post-KT.

With simultaneous consideration of tacrolimus blood concentration and the dose required to achieve the level of exposure, patients with increased risk of poor short- and long-term outcomes after KT can be identified. In a previous study, an association between a high early tacrolimus dose requirement and a significantly reduced kidney graft function had been reported^39^. A higher risk of having an acute rejection episode after transplantation has also been observed in KTRs in the high estimated tacrolimus clearance group compared with the low clearance group^40^. Furthermore, the low tacrolimus concentration/daily dose ratio has been associated with long term outcomes, including inferior graft function, death-censored graft loss risk, and higher mortality rate^41–43^. Tacrolimus pharmacokinetics is known to be influenced by various factors^44^. Some factors may affect tacrolimus exposure in the early phase while others exert its influence on the drug exposure in the late phase after transplantation. Whether or not the effect of diltiazem on increasing tacrolimus exposure would also improve short- and long-term clinical outcomes remains to be explored.

Hypertension (HTN) is one of the most common comorbidities after KT. HTN is an established risk factor for cardiovascular disease (CVD), the leading cause of death and graft loss in KTRs^45–47^. The pathogenesis of post-transplant hypertension is complex and different over time periods during which hypertension develops after KT^48^. Given that an adequate blood pressure and volume status is necessary to establish good kidney graft function, over-aggressive blood pressure lowering intervention should be avoided to minimize the risk of hypotension, although uncertainty remains over the optimal blood pressure control, especially during the early post-KT period. No hypotensive episode and no significant differences in blood pressures were observed among the study groups.

The present study has limitations that should be considered. First, albeit appropriate statistical calculations, the number of patients in the present study is quite small. Although the participants are randomly assigned, with allocation concealment, into groups, confounding bias may possibly exist. Further well-designed larger studies with longer follow up periods are needed to substantiate the findings. Second, all the KTRs who participated in this study received a combination of immediate release tacrolimus together with mycophenolate and corticosteroids; whether the effect of diltiazem significantly impacts tacrolimus exposure among KTRs who receive extended-release tacrolimus, steroid avoidance, or steroid withdrawal regimens is questionable. Third, this study is a single-center study; however, the study patients were uniformly treated with the same strategies for caring the patients post-KT. Finally, this study focused on tacrolimus levels during the first week post-KT; the clinical outcomes were not evaluated. Given that extensive therapeutic drug monitoring was performed and rapidly corrected any extreme tacrolimus concentrations and our study patients are low or standard risk KTRs, the under- or over-exposure of tacrolimus is not long enough to cause a clinically relevant increase in the incidence of concentration-related unwanted events during the study period. Consequently, the question of whether early achievement of target tacrolimus concentrations after KT is beneficial for KTRs with a high immunologic risk especially in ethnic populations with high prevalence of CYP3A5 expresser status remains to be answered.

## Conclusion

The very low dosage of 60 mg/day diltiazem affects tacrolimus exposure in CYP3A5 expressers. As compared with CYP3A5 nonexpressers, CYP3A5 expressers who did not receive the diltiazem coadministration had significantly lower AUC/D of tacrolimus at day 7 post-KT while CYP3A5 expressers who received the diltiazem coadministration did not. No significant effects on blood pressure were detected in the study patients.

Coadministration of the very low dose diltiazem could be considered as a possible strategy to increase exposure of tacrolimus early after KT, especially for CYP3A5 expressers.

## Data Availability

The datasets generated and/or analysed during the current study are available in the NCBI ClinVar repository (https://submit.ncbi.nlm.nih.gov/subs/variation_clinvar/accession number SCV002500936).

## References

[1] Wallemacq P (2009). Opportunities to optimize tacrolimus therapy in solid organ transplantation: Report of the European consensus conference. Ther. Drug Monit..

[2] Ekberg H (2007). Reduced exposure to calcineurin inhibitors in renal transplantation. N. Engl. J. Med..

[3] Dugast E (2016). Failure of calcineurin inhibitor (tacrolimus) weaning randomized trial in long-term stable kidney transplant recipients. Am. J. Transplant..

[4] Hricik DE (2015). Adverse outcomes of tacrolimus withdrawal in immune–quiescent kidney transplant recipients. J. Am. Soc. Nephrol..

[5] Oberbauer R (2020). Optimization of tacrolimus in kidney transplantation: New pharmacokinetic perspectives. Transplant. Rev..

[6] Zwart TC (2021). Model-informed precision dosing to optimise immunosuppressive therapy in renal transplantation. Drug Discov. Today.

[7] Andrews LM (2017). Pharmacokinetic considerations related to therapeutic drug monitoring of tacrolimus in kidney transplant patients. Expert Opin. Drug Metab. Toxicol..

[8] Staatz CE, Goodman LK, Tett SE (2010). Effect of *CYP3A* and *ABCB1* single nucleotide polymorphisms on the pharmacokinetics and pharmacodynamics of calcineurin inhibitors: Part I. Clin. Pharmacokinet..

[9] Phupradit A (2018). Impact of *POR* and *CYP3A5* polymorphisms on trough concentration to dose ratio of tacrolimus in the early post-operative period following kidney transplantation. Ther. Drug Monit..

[10] Hesselink DA, Bouamar R, Elens L, van Schaik RHN, van Gelder T (2014). The role of pharmacogenetics in the disposition of and response to tacrolimus in solid organ transplantation. Clin. Pharmacokinet..

[11] Undre NA (1999). Low systemic exposure to tacrolimus correlates with acute rejection. Transplant. Proc..

[12] Borobia AM (2009). Trough tacrolimus concentrations in the first week after kidney transplantation are related to acute rejection. Ther. Drug Monit..

[13] O’Seaghdha CM (2009). Higher tacrolimus trough levels on days 2–5 post-renal transplant are associated with reduced rates of acute rejection. Clin. Transplant..

[14] Richards KR (2014). Tacrolimus trough level at discharge predicts acute rejection in moderately sensitized renal transplant recipients. Transplantation.

[15] MacPhee IA (2004). The influence of pharmacogenetics on the time to achieve target tacrolimus concentrations after kidney transplantation. Am. J. Transplant..

[16] Jones DR (1999). Diltiazem inhibition of cytochrome P-450 3A activity is due to metabolite intermediate complex formation. J. Pharmacol. Exp. Ther..

[17] Cornwell MM, Pastan I, Gottesman MM (1987). Certain calcium channel blockers bind specifically to multidrug-resistant human KB carcinoma membrane vesicles and inhibit drug binding to P-glycoprotein. J. Biol. Chem..

[18] Kidney disease: improving global outcomes transplant work group. KDIGO clinical practice guideline for the care of kidney transplant recipients. *Am. J. Transplant*. **9**, S1–S155 (2009). 10.1111/j.1600-6143.2009.02834.x 19845597

[19] Li JL (2011). Effects of diltiazem on pharmacokinetics of tacrolimus in relation to *CYP3A5* genotype status in renal recipients: From retrospective to prospective. Pharmacogenomics J..

[20] ARCHITECT tacrolimus Ref1L77. Abbott Park: Abbott Laboratories Division (2015).

[21] Kuehl P (2001). Sequence diversity in *CYP3A* promoters and characterization of the genetic basis of polymorphic CYP3A5 expression. Nat. Genet..

[22] MacPhee IA, Holt DW (2008). A pharmacogenetic strategy for immunosuppression based on the *CYP3A5* genotype. Transplantation.

[23] Vannaprasaht S (2013). Personalized tacrolimus doses determined by *CYP3A5* genotype for induction and maintenance phases of kidney transplantation. Clin. Ther..

[24] Yaowakulpatana K (2016). Impact of *CYP3A5* polymorphism on trough concentrations and outcomes of tacrolimus minimization during the early period after kidney transplantation. Eur. J. Clin. Pharmacol..

[25] Chen L, Prasad GVR (2018). *CYP3A5* polymorphisms in renal transplant recipients: Influence on tacrolimus treatment. Pharmgenomics Pers. Med..

[26] Park SY, Kang YS, Jeong MS, Yoon HK, Han KO (2008). Frequencies of *CYP3A5* genotypes and haplotypes in a Korean population. J. Clin. Pharm. Ther..

[27] Supanya D (2009). Prevalence of *CYP3A5* polymorphism in a Thai population. Thai. J. Pharmacol..

[28] Balram C, Zhou Q, Cheung YB, Lee EJ (2003). *CYP3A5*3* and **6* single nucleotide polymorphisms in three distinct Asian populations. Eur J Clin Pharmacol..

[29] Zhao P, Lee CA, Kunze KL (2007). Sequential metabolism is responsible for diltiazem-induced time-dependent loss of CYP3A. Drug Metab. Dispos..

[30] Zhang X, Quinney SK, Gorski JC, Jones DR, Hall SD (2009). Semiphysiologically based pharmacokinetic models for the inhibition of midazolam clearance by diltiazem and its major metabolite. Drug Metab. Dispos..

[31] Sutton D, Butler AM, Nadin L, Murray M (1997). Role of CYP3A4 in human hepatic diltiazem *N*-demethylation:inhibition of CYP3A4 activity by oxidized diltiazem metabolites. J. Pharmacol. Exp. Ther..

[32] Mayhew BS, Jones DR, Hall SD (2000). An in vitro model for predicting in vivo inhibition of cytochrome P450 3A4 by metabolic intermediate complex formation. Drug Metab. Dispos..

[33] Ma B, Prueksaritanont T, Lin JH (2000). Drug interactions with calcium channel blockers: Possible involvement of metabolite-intermediate complexation with CYP3A. Drug Metab. Dispos..

[34] Pinto AG (2005). Diltiazem inhibits human intestinal cytochrome P450 3A (CYP3A) activity in vivo without altering the expression of intestinal mRNA or protein. Br. J. Clin. Pharmacol..

[35] Pauli-Magnus C, Kroetz DL (2004). Functional implications of genetic polymorphisms in the multidrug resistance gene. Pharm. Res..

[36] Christians U (2006). Active drug transport of immuno-suppressants: New insights for pharmacokinetics and pharmacodynamics. Ther. Drug. Monit..

[37] Jones TE, Morris RG (2002). Pharmacokinetic interaction between tacrolimus and diltiazem: Dose–response relationship in kidney and liver transplant recipients. Clin. Pharmacokinet..

[38] Birdwell KA (2015). Clinical pharmacogenetics implementation consortium (CPIC) guidelines for *CYP3A5* genotype and tacrolimus dosing. Clin. Pharmacol. Ther..

[39] Kuypers DRJ (2010). Tacrolimus dose requirements and *CYP3A5* genotype and the development of calcineurin inhibitor-associated nephrotoxicity in renal allograft recipients. Ther. Drug Monit..

[40] Egeland EJ (2017). High tacrolimus clearance is a risk factor for acute rejection in the early phase after renal transplantation. Transplantation.

[41] Thölking G (2014). The tacrolimus metabolism rate influences renal function after kidney transplantation. PLoS ONE.

[42] Jouve T (2020). The TOMATO study (tacrolimus metabolization in kidney transplantation): Impact of the concentration–dose ratio on death-censored graft survival. Transplantation.

[43] Schütte-Nütgen K (2019). Fast tac metabolizers at risk—It is time for a C/D ratio calculation. J. Clin. Med..

[44] Staatz CE, Tett SE (2004). Clinical pharmacokinetics and pharmacodynamics of tacrolimus in solid organ transplantation. Clin. Pharmacokinet..

[45] Kidney disease: improving global outcomes (KDIGO) blood pressure work group. KDIGO clinical practice guideline for the management of blood pressure in chronic kidney disease. *Kidney Int., Suppl*. **99**, S1–S87 (2021). 10.1016/j.kint.2020.11.003 33637192

[46] Ojo AO (2006). Cardiovascular complications after renal transplantation and their prevention. Transplantation.

[47] Kasiske BL (2001). Epidemiology of cardiovascular disease after renal transplantation. Transplantation.

[48] Tantisattamo E (2020). Approach and management of hypertension after kidney transplantation. Front. Med..

